# ASSOCIATION BETWEEN CARDIOVASCULAR DISEASE AND COGNITIVE IMPAIRMENT: IS DEPRESSION A MEDIATOR?

**DOI:** 10.1093/geroni/igad104.2938

**Published:** 2023-12-21

**Authors:** Benita Bamgbade, James Lovato, David Reboussin, Stephen Rapp, Byron Jaeger, Jeff Williamson

**Affiliations:** Northeastern University, Boston, Massachusetts, United States; Wake Forest School of Medicine, Winston Salem, North Carolina, United States; Wake Forest School of Medicine, Winston Salem, North Carolina, United States; Wake Forest School of Medicine, Winston Salem, North Carolina, United States; Wake Forest School of Medicine, Winston Salem, North Carolina, United States; Wake Forest School of Medicine, Winston Salem, North Carolina, United States

## Abstract

By 2040, over 9 million US adults will live with dementia. Understanding the relationship between risk factors and cognitive decline is critical to prevent disease and slow progression. Depression and cardiovascular disease (CVD) are independent risk factors for cognitive impairment (CI) which may interact, suggesting depression may play a mediating role in the relationship between CVD and CI, an hypothesis explored in this study. Data from the Systolic Blood Pressure Intervention Trial (SPRINT), a multi-center, randomized, controlled trial were used in this multivariate logistic regression analysis. CI was defined as adjudicated cases of mild CI and probable dementia. Depression was defined as a Patient Health Questionnaire-9 score ≥10 or current antidepressant therapy. We developed a CVD risk score using data from SPRINT participants and discretized the scores into tertiles. Of 9259 SPRINT participants, our analytic sample included 8456 who had adjudicated mild CI or dementia and had baseline depression scores. They were on average 68 years and 36% were female. After adjusting for age, sex, education, race, prior CVD, chronic kidney disease and treatment assignment, participants with high cardiovascular risk were more likely to have CI (OR: 1.55, 95% CI: 1.17-2.05). Participants with depression were more likely to have CI (OR: 1.29, 95% CI: 1.05-1.58) however, the interaction between cardiovascular risk and cognition was not significant. Findings from this study suggest that while depression and CVD are associated with CI, depression may not explain the CVD-CI relationship. More research is needed to understand the relationship between depression, CVD and CI.

